# CIRSE Standards of Practice on Oesophageal and Gastroduodenal Stenting

**DOI:** 10.1007/s00270-023-03395-0

**Published:** 2023-03-14

**Authors:** Athanasios Diamantopoulos, Shuvro Roy Choudhury, Farah Gillian Irani, Hugo Rio Tinto, Tarun Sabharwal

**Affiliations:** 1 Department of Interventional Radiology, Guy’s and St. Thomas’ NHS Foundation Trust, St Thomas’ Hospital, London, UK; 2 School of Biomedical Engineering & Imaging Sciences, Faculty of Life Sciences & Medicine, Kings College London, London, UK; 3 Radiology, Rabindranath Tagore International Institute of Cardiac Sciences, Kolkata, India; 4 Department of Vascular and Interventional Radiology, Singapore General Hospital, Singapore, Singapore; 5 Radiology Department, Champalimaud Foundation, Lisbon, Portugal

**Keywords:** Self-expanding metal stents, Gastrointestinal, Oesophageal, Gastroduodenal, Radiology, Interventional radiology

## Abstract

**Background:**

Image-guided insertion of stents in the upper gastrointestinal trunk is an effective, minimally invasive treatment option to provide immediate relief of symptoms caused by upper gastrointestinal tract obstruction related to advanced-stage malignant causes or benign causes that lead to lumen narrowing.

**Purpose:**

This document, as with all CIRSE Standards of Practice documents, is not intended to impose a standard of clinical patient care but will recommend a reasonable approach to best practices for performing stenting of the upper gastrointestinal tract, namely the oesophageal and gastroduodenal segments. Our purpose is to provide up-to-date recommendations for placement of upper gastrointestinal tract stents based on the previously published guidelines on this topic in 2005 and 2007.

**Methods:**

The writing group was established by the CIRSE Standards of Practice Committee and consisted of a group of internationally recognised experts in performing upper gastrointestinal stenting. The writing group reviewed the existing literature using PubMed to search for relevant publications in the English language up to September 2021. The final recommendations were formulated through consensus.

**Conclusion:**

Insertion of stents in the oesophageal and gastroduodenal tracts has an established role in the successful management of malignant or benign obstructions. This Standards of Practice document provides up-to-date recommendations for the safe performance of upper gastrointestinal stent placement.

## Introduction

We aim to present an update of the previous CIRSE Standards of Practice (SOP) documents on upper gastrointestinal (GI) stenting with a focus on contemporary practice and emphasis on the indications, absolute and relative contraindications, available stent designs, technical and clinical outcomes, and complications. This CIRSE Standards of Practice document is not intended to impose a standard of clinical patient care but recommends a reasonable approach to best practices for placing self-expanding metal stents (SEMS). Institutions should regularly review their internal procedures, taking into account international guidance, local resources and regular internal morbidity and mortality reviews.

## Methods

The CIRSE Standards of Practice Committee established a writing group that was tasked with producing up-to-date recommendations for performing upper gastrointestinal stent procedures.

The writing group consisted of clinicians with internationally recognised expertise in performing upper gastrointestinal tract stenting. The writing group reviewed existing literature on upper gastrointestinal stenting, performing a pragmatic evidence search using PubMed for relevant publications in the English language up to September 2021.

## Background

Self-expanding metal stents are commonly used worldwide in order to give immediate relief of dysphagia symptoms caused by upper gastrointestinal tract obstruction related to advanced-stage oesophageal cancer, gastroduodenal cancers or other malignant or benign causes that lead to lumen narrowing [1–4]. The CIRSE Standards of Practice Committee established a writing group tasked with the production of up-to-date recommendations for placement of upper gastrointestinal tract stents based on the previously published guidelines on this topic in 2005 and 2007 [3, 5].

The main indications are a) palliation of symptomatic obstruction caused by either endoluminal or extrinsic malignant causes; b) management of perforations with or without the development of fistulae; c) management of anastomotic leaks or local recurrences; and finally, d) symptom relief for benign strictures resistant to repeat balloon dilatation. In patients with malignant obstruction, many of whom are not candidates for curative surgical resection at first presentation due to advanced disease, stents have proven vital in supporting nutritional intake and thus significantly improving quality of life [2, 3].

## Pre-Procedural Patient Assessment, Preparation and Planning

Each patient should be discussed at a multidisciplinary team meeting consisting of gastroenterologists, upper gastrointestinal surgeons, interventional radiologists (IRs) and oncologists for individualised, consensus-based, best-option treatment plans after reviewing all the available clinical notes, clinical presentation, pre-existing diagnostic examinations (endoscopic and radiologic studies) [3, 5], functional status and expected subsequent therapies [6].

Appropriate imaging is essential as it provides the appropriate information regarding the disease extent, the lesion site as well as the presence of any disease distally that may affect the decision or the procedure outcome, as further obstructions may compromise the passage of the enteric content [5]. Normally, a contrast-enhanced computer tomography (CT) scan of the chest and abdomen should suffice. In case of fistulae or leaks, a CT study with simultaneous water-soluble contrast or a fluoroscopic water-soluble swallow study may be required to define the defect. Specifically, in case of upper oesophageal tumours, particular attention needs to be given to the upper extent of the tumour. In general, obstructions above C7 are difficult to manage owing to the proximity of the cricopharyngeus or upper oesophageal sphincter.

It is essential that the interventional radiologist should consult with the patient, present the plan and the alternative options, if any, and obtain written informed consent prior to the procedure. Appropriate laboratory work-up is recommended [7]. In cases of gastroduodenal obstruction, it is useful to insert a nasogastric tube to decompress the stomach prior to the procedure. Finally, the need for anaesthetic support or nurse-led sedation provided by a trained member of the IR team is determined during pre-procedural assessment. Patients on anticoagulation or antiplatelets need these to be suspended prior to procedure as per the CIRSE Standards of Practice [7, 8].

## Oesophageal Stenting

### Indications and Contraindications for Treatment

#### Indications

The main benefit of oesophageal stent placement is relief from obstructive symptoms allowing diet resumption leading to improved quality of life. The symptoms can be due to primary or metastatic oesophageal malignancy or extrinsic tumour compression (i.e. lung or mediastinal masses).

Other indications include:

- Malignant perforation with or without tracheo-oesophageal fistula
- Iatrogenic perforation
- Surgical anastomotic leaks
- Local recurrence following surgery
- Benign refractory strictures [3].

Unusual indications include a leak following sleeve gastrectomy in bariatric surgery and to treat recurrent emergent variceal bleeding after failed endoscopy as a bridge to more definitive therapy. A full list of the indications and contraindications is shown in Table 1.

**Table 1:** Oesophageal stenting (indications and contraindications)

Indications	Contraindications
Malignant oesophageal strictures causing obstruction	Treatable disease
Tracheo-oesophageal fistulae	Uncorrectable coagulopathy (please refer to CIRSE guidelines [7])
Mediastinum tumours (primary or secondary) causing extrinsic compression	Very proximal lesion (within 2 cm from the upper oesophageal sphincter)
Oesophageal perforation	Risk of airway compression
Symptomatic malignant gastro-oesophageal anastomotic leaks	Recent (within 6 weeks) high-dose chemo-/radiotherapy
Post-surgery anastomotic tumour recurrence	Limited life expectancy (terminally ill patients)
Benign oesophageal strictures resistant to balloon dilatation and unsuitable for surgery	Distal obstruction (gastroduodenal and/or small bowel)
Leak following bariatric surgery	Confirmed sepsis

#### Contraindications

As the main reason for stenting is dysphagia, stents should not be used in asymptomatic patients who can tolerate feeding. Stenting should also be avoided in patients with potentially curable disease. In some instances, removable stents could be placed to facilitate enteral feeding during chemotherapy treatment given to downstage a tumour prior to possible curative surgery. Routine use of oesophageal stents in place of other enteral feeding methods to improve or maintain nutrition is not recommended in the neoadjuvant setting [8].

Other contraindications to oesophageal stenting include:

- Uncorrectable coagulopathy
- Serious risk of airway obstruction if a preventative tracheal stent cannot be inserted concurrently
- Evidence of distal gastroduodenal or small bowel obstruction (relative contraindication)
- Presence of free peritoneal perforations (relative contraindication)
- Recent high dose of systemic chemotherapy (as this may lead to increased haemorrhage and perforation rates) (relative contraindication)
- Terminally ill patients [3]

### Treatment/Technique

Prior to the procedure, the site and length of the stricture are determined by an oesophagogram or pre-procedural CT scan. Stents are usually inserted in the IR suite, via a transoral approach, using fluoroscopic guidance, under either conscious sedation (midazolam and/or fentanyl) or general anaesthesia (GA) combined with topical anaesthesia of the pharynx with lidocaine spray.

The patient is usually placed in the left lateral decubitus position, or rarely, in the supine position. If possible, the bed is tilted with the head-end up to reduce the risk of aspiration.

Using standard angled-tip catheters (i.e. biliary manipulation or multipurpose catheters) and standard or hydrophilic wires, the stricture is crossed under fluoroscopic guidance into the stomach, the position of which is confirmed with injection of contrast. The hydrophilic wire is then replaced by a stiff 0.035″ support wire (i.e. Amplatz-type wire), the tip of which is either coiled within the stomach or placed in the duodenum to allow safe delivery of subsequent devices. Following this, an appropriate size (usually 5 or 6 Fr) and length (i.e. 55 cm) sheath are advanced past the stricture. The location of the tumour is confirmed by doing a pull-back oesophagogram through the sheath. The upper and lower ends of the stricture are defined and marked using anatomic landmarks. Subsequently, an appropriately sized stent is advanced across the stricture and deployed, making sure to cover the whole lesion with the ends of the stent landing in normal mucosa.

In the past, routine pre-dilation was not advisable because of the increased risk of perforation; however, a gentle pre-dilation using a 10–12-mm balloon is acceptable to allow stent delivery though a tight stenosis [1–3]. The stent length should accommodate at least 2 cm of normal oesophagus proximal and distal to the lesion. To minimise the possibility of the more common distal migration, care should be taken so more of the stent length is proximal to the stricture than distal to it, if possible. Should the lesion require more than one stent to cover the whole length, the two devices should overlap by at least a third.

Following completion of the procedure, another pull-back contrast study is performed to confirm correct stent deployment with full lesion coverage and to exclude any immediate complications such as perforation [1, 3, 9]. Contrast is also injected above the stent to confirm free drainage. Following completion, a chest X-ray is performed after 24 h to check for full stent opening. Although the stents gradually reach their nominal diameter in the first 2 days, if a covered stent is used, some operators suggest post-stent dilatation to allow immediate complete stent expansion [1, 2].

### Post-Treatment and Follow-Up Care

After successful stent deployment with no immediate complications, patients can start oral fluids 6 h post-procedure and continue with a liquid diet for the first 12–24 h, followed by low-residue diet, and gradually start more solid food. It is recommended to avoid large lumps of food, and patients are encouraged to drink liquids freely, especially carbonated drinks, during the day and especially with each meal [3]. All patients who develop reflux are advised to start an antacid, preferably a proton-pump inhibitor. Some operators recommend the empirical use of antacids if a non-valve stent is inserted across the gastro-oesophageal junction (GOJ) [1, 2].

### Outcome Measures

#### Technical Success and Clinical Outcomes

Technical success of stenting using fluoroscopic guidance is close to 100% [10].

The clinical outcomes are evaluated using the dysphagia score, which has five grades: Grade 0: patient can tolerate normal diet; Grade 1: can have some solid food; Grade 2: only semi-solid foods are tolerated; Grade 3: patients can only take liquids; Grade 4: complete dysphagia. Following stenting, dysphagia is relieved in the majority of patients with the score improving at least by one grade in up to 98% of patients [2, 3, 11–15].

Oesophageal stenting for malignant disease remains a palliative procedure with the aim of improving the patient’s quality of life [2, 9]. Clinical success (dysphagia symptom improvement) has been reported in 46.2% of cases during a 74-week median follow-up in recent published studies [16, 17]. Higher clinical success of around 96% has been reported at a median follow-up of 129 days in one series [10]. Primary median stent patency, where reported, ranges between 71 and 145 days [18, 19].

Reports of radioactive I-125-loaded metallic stents have shown promise in increasing stent patency. In a recent meta-analysis comparing these stents to conventional SEMS, these stents have shown similar dysphagia improvement rates and complication rates but significantly improved stent patency and survival [20].

In cases of malignant oesophago-respiratory fistulae, a technical success rate of 100% and an initial clinical success rate of 78.4% have been reported. Aspiration symptoms recurred in 37% with an overall complication rate of 25% [21]. In anastomotic leaks and/or perforations, the clinical success of covered metallic stents ranges between 95 and 100%, with the stents being removed after 6 weeks to 3 months [3, 22, 23]. A conformable knitted nitinol stent or a lumen-apposing metal stent (LAMS) may be superior in this regard [23].

Strictures and fistulae close to the cricopharyngeus are difficult to treat due to the higher risk of globus symptoms and aspiration after stenting. However, in one series of 104 patients within 8 cm of the cricopharyngeus, a technical success of 96%, average dysphagia score reduction by 2 points and a fistula-sealing rate of 79% have been achieved [24]. Dedicated stents with a short proximal flare or a narrower lumen can be used in this setting.

The application of stents for benign disease has increased considerably over the last decade. Self-expanding plastic stents (SEPS) are potentially attractive in this regard due to their ease of insertion, limited tissue reaction and ease of removal. However, they are prone to migration. Holm et al. reported 68.1%, 30% and 70.4% migration rates in proximal, mid- and distal strictures, respectively [25].

Primary clinical success for the management of benign strictures resistant to balloon dilatation is approximately 100%; however, these patients develop recurrent dysphagia due to hyperplasia, which can be treated with balloon dilatation or laser therapy [3]. In a series of 130 patients with benign oesophageal strictures, after a median follow-up of 13 months, only 52% of patients were dysphagia-free, the early migration rate was 24%, and major clinical complications occurred in 9% [26].

Given the long-term problems with SEPS, some authors have advocated for the use of fully covered SEMS for benign strictures and leaks. In a series of 31 patients with benign pathologies, 28 (90.3%) reported dysphagia relief, while complications were reported in 13 patients (11 cases of migration and 2 cases of chest pain). Forty-one out of forty-three stents placed in this series were successfully removed [27].

Conventional covered SEMS and customised stents have also been used to treat leaks following bariatric surgery, particularly sleeve gastrectomy. These are often long stents, and there is some low-level evidence to suggest that oesophagoduodenal megastents may be better in this regard [28].

#### Complications and Management

Complications can be divided into procedural-related (early) and post-procedural (delayed, beyond 2 weeks) complications.

Procedural-related complications include oesophageal perforation and/or fistula, aspiration, fever, globus sensation, haemorrhage, stent migration, reflux symptoms and post-procedural chest pain. In one series, early complications were reported in up to 32% of cases, with stent migration being the most common complication. Oesophageal stenting carries a low mortality rate of 0–1.4% [1–3, 29]. Major complications are listed in Table 2. Procedural complications are less common with metallic stents as compared to rigid plastic endoprosthesis [3]. Post-procedural chest pain is quite common in the early post-implantation period; however, prolonged pain is only seen in less than 14% of cases [2, 16]. Pain is more severe when using stents with larger diameters and treating proximal strictures [3].

**Table 2:** Major complications associated with oesophageal stents

Complication	Frequency
Haemorrhage	3–8%
Chest pain	14%
*Stent migration*	
Uncovered stent	0–6%
Covered stent	25–32%
*Tumour ingrowth*	
Uncovered stent	17–36%
Covered stent	Negligible
Fistula and/or perforation	Uncommon
Death	< 1.4%

Later complications include: perforation; haemorrhage, which in the majority of cases is self-limited but very rarely requires embolisation; stent migration; pain and/or sensation of a foreign body; restenosis or reocclusion due to either tumour ingrowth or overgrowth; occlusion as a result of food bolus; oesophagitis; reflux; fever; fistula formation; airway compromise; and sepsis. In one series of 133 patients, Homann et al. reported a delayed complication rate of 53.4%. Recurrent dysphagia was due to tumour ingrowth (22%), bolus obstruction (21%), stent migration (9%) and respiratory fistulae (9%) [30].

Uncovered stents migrate less (between 0 and 3%) compared to covered stents, and the incidence of migration is higher if stents are positioned across the cardia (6% for uncovered and 32% for covered stents) [2, 3, 16, 29]. One meta-analysis using covered SEMS reported a 26.4% migration rate at a mean of 17 days post-insertion [16, 17]. Further recent studies using fully covered stents reported an overall complication rate of 39.5%, the most common of which is migration (36.3%) [19]. Partially migrated stents within the oesophagus are managed by coaxial insertion of an additional stent with significant overlap. Completely migrated stents are managed with a new stent with or without endoscopic removal of the previous stent from the stomach [3].

Re-obstruction due to ingrowth ranges between 17 and 36% for uncovered stents and is uncommon for covered stents [2, 3, 16]. Adverse events, including tumour overgrowth, may occur in up to 60% of the patients who are followed up for long periods [3, 31].

Insertion of a new stent can successfully manage symptoms of both tumour ingrowth and overgrowth. Cases of dysphagia caused by epithelial hyperplasia or granulation can be treated using several techniques such as laser, argon beam treatment and restenting [3]. Rarer reported complications include bowel obstruction due to stent migration, oesophago-pericardial fistula, etc. [1, 32, 33].

### Available Oesophageal Stent Designs

Stents may be made of plastic (SEPS) or metal (SEMS), the latter being made of stainless steel or nitinol, and may be fully covered, partly covered or uncovered. Covered SEMS are the primary choice of stent for the management of malignant oesophageal lesions, with their main advantage being the avoidance of tumour ingrowth [1–3]. With the improved design of the available devices, including flaring of the proximal segment, dog bone designs, segments of partly uncovered stents and the covering material being on the inside, the previous high risk of migration is now limited [1–3, 16]. Further techniques to prevent migration include anchoring sutures or clips if delivered at endoscopy. Covered oesophageal stents are also the first choice in cases of tracheo-oesophageal fistulae and leaks as a result of perforation.

Uncovered SEM stents are reserved for cases with extrinsic compression, massively dilated oesophagus to minimise the possibility of food being trapped between the stent and the oesophageal wall, or cases with previous gastric pull-up. However, these are associated with higher risks of bleeding, fistulae, tumour ingrowth and embedment [34]. Stents with short proximal flares are available for strictures or leaks in the cervical oesophagus, although these should be used with caution [1–3, 16].

Stents with anti-regurgitation valves are used to prevent gastric acid reflux when stents are placed across the gastroesophageal junction, although some studies claim that the appropriate use of proton-pump inhibitor therapy has the same results. Retrievable stents should be considered in the treatment of benign disease and in patients with cancer who are suitable for surgery, but suffer from significant dysphagia and are in need of nutrition and weight gain prior to operation [1–3, 16].

Most of the currently available stents are retrievable or adjustable, either due to their design by pulling a lasso string or wire attached at one or both ends, or by using a dedicated extractor device before full endothelialisation, usually at endoscopy. Fluoroscopic techniques using a hook wire and a 13-Fr oversheath are also described, either by collapsing the head of the stent by pulling, or by everting the stent by hooking and pulling the distal wire loop [35].

Current experience with biodegradable stents is limited, and it is mainly in the management of the benign strictures. They can be composed of polydioxanone, a surgical suture material (ELLA-CS, Hradec Králové, Czech Republic) or poly-L-lactic acid (PLLA) knitted monofilaments (Marui Textile Machinery, Osaka, Japan: not currently available in Europe) and work on the principle of degradation by gradual hydrolysis after implantation. Most reports demonstrate non-superiority and report similar/higher incidence of adverse events [36, 37]. A meta-analysis of 18 studies with 444 patients compared the results of fully covered SEMS, SEPS and biodegradable stents in benign refractory oesophageal strictures; clinical success rates of 40.1, 46.2 and 32.9% (*p* = not significant), respectively, were reported. No difference in the pooled migration rate (28.6%) and the overall complication rate (20.6%) was noted [38].

The ideal stent, therefore, remains elusive. Ongoing and future research areas in stent design include 3D-printed stents and stents with chemotherapeutic drug elution or radioactive bead-loaded stents.

## Gastroduodenal Stenting

### Indications and Contraindications for Treatment

#### Indications

Patients with gastroduodenal obstruction present with nausea, vomiting, weight loss, malnutrition and dehydration secondary to poor oral intake leading to very poor quality of life, and they can benefit from gastroduodenal stenting.

The main indications for gastroduodenal stenting are:

- Non-operable or untreatable disease due to:
  - a) Intrinsic tumours (gastric or duodenal cancers)—patients usually present quite late with up to 40% being inoperable at the time of diagnosis;
  - b) Extrinsic compression causing luminal narrowing mainly due to pancreatic cancer and less commonly from cholangiocarcinoma, malignant lymphadenopathy, localised intraperitoneal metastasis or lymphoma [1, 5, 39–41].
- Disease recurrence at the anastomotic site following palliative or curative upper gastrointestinal surgery [1, 5, 39–42].
- Pyloric obstruction and dysfunction after gastric pull-up surgery when repeated dilatations failed to control symptoms [5, 43].
- Management of malignant fistulas using covered stents [5, 44, 45].
- Non-surgical patients with benign strictures due to chronic ulcer disease following failed balloon dilatation [5, 39, 45–47].

#### Contraindications

In general, patients that are considered for stent placement usually have a short life expectancy (less than 6 months) [5, 16, 45, 48, 49].

Contraindications for the procedure include:

- Perforation resulting in peritonitis
- Sepsis
- Extensive peritoneal carcinomatosis
- Distal small bowel obstruction
- Bowel ischaemia
- Uncorrectable coagulopathy

#### Treatment Options

Open surgical gastrojejunostomy has been used for palliation in these patients but carries a higher surgical risk and longer hospital stay, although the bypass tends to remain open longer with fewer reinterventions. Laparoscopic gastrojejunostomy is minimally invasive but still requires general anaesthesia and carries surgical risks in this fragile group of patients [50].

Endoscopic ultrasound-guided gastrojejunostomy (EUS GJ) using short lumen-apposing metal stents (LAMS) is a recently developed minimally invasive procedure which obviates the surgical risks. However, it requires high technical skill, is not widely available (outside of high-volume centres) and can be complicated by stent malpositioning [51].

Placement of enteral stents (endoscopically or radiologically) is a minimally invasive, safe procedure providing fast and effective relief of symptoms with short hospital stay, although duration of patency is shorter, requiring additional procedures.

### Treatment/Technique

The usual access for insertion of gastroduodenal stents is the transoral route, although alternative routes through a percutaneous gastrostomy have been described.

Crossing, positioning and stent deployment are guided by either fluoroscopy alone or in combination with endoscopy [1, 5, 16, 45, 49]. Fluoroscopy is essential as it allows accurate stent positioning and deployment [1, 5, 52]. The procedure can be performed under either conscious sedation or general anaesthesia transorally [1, 5, 53].

The day prior to the procedure, a nasogastric tube should be inserted and kept on low wall suction to help decompress the stomach in order to allow easier negotiation of the stomach and avoid catheter guidewire redundancy within the dilated stomach. Patients are placed in the lateral decubitus position, or rarely, in the supine position with reverse Trendelenburg to reduce the risk of aspiration. The pharynx is anaesthetised using topical lidocaine spray. An appropriate size angled-tip catheter (90–100 cm, usually 5- or 6-Fr multipurpose catheter) and hydrophilic wire are manipulated to just proximal to the stricture, and by standard wire/catheter technique, the stricture is crossed. Following successful crossing, double-contrast duodenography (using contrast and air) is performed to delineate the distal end of the stricture and confirm position in normal healthy duodenum. In cases of recurrent looping of the catheter guidewire system in the stomach, a large long sheath (i.e. 11-Fr sheath) can assist in overcoming the problem [5, 53].

Once the catheter is positioned beyond the stricture in the proximal jejunum, a 260-cm exchange length (or longer, up to 450 cm) stiff wire is inserted to assist with stent delivery, especially with long delivery systems. Prior to stent deployment, a long sheath is advanced across the stricture and the lesion is delineated using pull-back duodenography.

An appropriately sized duodenal stent (minimum diameter 18 mm and appropriate length at least 4 cm longer than the length of the stricture) is deployed under fluoroscopic guidance [5, 52, 53]. Longer stents are preferred as they conform better to the curvature of the duodenum, avoiding kinking and allowing full coverage of the stricture [1, 5, 39]. Pre-dilatation can be necessary in cases of very tight stenosis; however, this needs to be performed with caution due to the associated risk of perforation. Post-dilatation could be an option in order to achieve maximum immediate lumen gain, but this should be reserved for cases where a covered stent has been used; for non-covered stents, there is an associated risk of perforation. Generally, self-expanding stents reach their full diameter within 24 h [1, 5, 39, 47, 53, 54].

In cases where more than one stent needs to be deployed to allow full lesion coverage, the distal stent should be deployed first in a straight segment of duodenum to reduce the possibility of obstruction or erosion of the bowel wall. The stents need to overlap adequately (at least 2–3 cm) to reduce the chance of separation due to bowel peristalsis [5, 44, 45, 48, 49]. Post-stent deployment, contrast is injected to assess appropriate position and exclude perforation.

In some cases, support from an endoscope can be useful, especially when the catheter/guidewire combination constantly coils in the stomach [1, 5, 49, 52]. In addition, endoscopy can assist with direct visualisation of the stricture, which is then crossed using a hybrid combination of fluoroscopy and direct vision through the endoscope. Once the lesion is crossed, the stent is always deployed under continuous fluoroscopic guidance to ensure accurate placement and stent deployment [1, 5]. In cases where, despite endoscopic assistance, it is not possible to cross the stricture due to redundancy in the stomach, an alternative approach through a shorter direct route via a pre-existing or newly formed percutaneous gastrostomy could be utilised [1, 5].

In patients presenting with obstructive jaundice, in whom the gastroduodenal stricture has extended to involve the ampulla of Vater, simultaneous insertion of both biliary and duodenal stents should be considered [5, 55]. The biliary stent should be deployed before the duodenal stent to ensure that the stents lie side by side in the duodenal lumen [5, 54]. An alternative approach could be for the biliary stent to be deployed through the mesh of the duodenal stent [41, 48].

### Post-Treatment and Follow-Up Care

Following gastroduodenal stent deployment, patients remain nil by mouth for the first 6 h. They are continuously monitored for any signs of peritonitis. Clear fluids can be started after 6 h followed by liquid diet for the next 12–24 h, progressing to a soft diet, and eventually starting a solid diet with the aid of an appropriately trained dietician. They are advised to take small mouthfuls, eat slowly and chew their food well. Sauces and/or butter can be used to moisten food. Intake of carbonated drinks at meals is additionally recommended to help maintain stent patency [1, 5, 52]. Some operators still recommend an upper gastrointestinal study with water-soluble contrast medium to assess stent position, patency and adequacy of lesion coverage. The study is deemed complete once contrast is visualised distal to the stent without evidence of contrast extravasation [5].

Following discharge, patients are reviewed in clinic, where additional imaging is requested for patients who become symptomatic or develop complications.

### Outcome Measures

Table 3 summarises the published results on various outcome measures.

**Table 3:** Summary of published results on various outcome measures with gastroduodenal stenting

Criterion	Results
Technical success	94–97%
Clinical success	71–91%
Time to final resolution of symptoms	3.0–3.7 days
Hospital-free survival	4–13 weeks

#### Technical Success

Technical success is defined as the ability to cross a stricture and successfully deploy a stent at the site of the narrowing. The procedure is associated with high technical success rates [5, 16, 53, 56]. A small number of cases are not successful due to complex and/or distorted anatomy, high-grade stenosis or looping within a dilated stomach. As a result, these patients may have to undergo surgical bypass [5, 16, 41, 56, 57].

#### Clinical Success

Clinical success is defined as either resolution or improvement of symptoms post-stent deployment [5, 16]. Up to 90% of patients can tolerate oral food one day post-procedure [16, 53, 58]. In cases in which this is achieved, the median stent patency has been calculated to be 279 days [58]. In a cohort of patients, despite technical success, they may not experience symptom relief and continue to remain malnourished. In part, this may be due to the lack of propulsive peristalsis in a chronically obstructed stomach, undiagnosed distal small-bowel strictures or functional gastric outlet obstruction (GOO) from neural (celiac axis) tumour involvement [1, 5, 16, 41, 46, 53, 59].

#### Hospital-Free Survival

Patients are generally discharged home or to a hospice within a few days of stent deployment. Following successful progression to soft or solid diets, patients can lead relatively independent lives [5, 60]. They are followed up in clinic and advised to seek medical attention if they become symptomatic prior to review.

#### Complications

The reported morbidity rate ranges between 11 and 43% [16, 61]. The most common complication is stent obstruction. Food bolus, tumour ingrowth and tumour outgrowth are responsible for the majority of cases [5], with patients developing symptom recurrence requiring endoscopic examination. Patients with tumour ingrowth or overgrowth may be suitable for coaxial insertion of an additional stent. Patency rates for secondary stents range between 80 and 100% [5, 39].

Perforation is a major life-threatening complication which may require urgent surgical intervention. Early perforation (< 24 h) could be the result of either guidewire manipulation (usually without sequelae) or balloon dilatation (which often needs surgery) [1, 5, 39]. Late perforation (> 24 h) is caused by stent erosion through the intestinal wall [5, 41, 42, 45].

Haemorrhage, in the majority of cases, is minor and managed conservatively [5, 62]. Patients with large exophytic vascular tumours are more vulnerable to life-threatening haemorrhage, as stenting can cause ulceration by pressure necrosis. In these cases, vascular embolisation may be required [5].

Stent migration is more often associated with covered stents [5, 60]. Proximally migrated stents can be retrieved endoscopically, whereas distally migrated stents can be managed conservatively if non-obstructing [5, 40]. They have occasionally been passed per rectum with no sequelae [5, 41, 59]. If, however, their migration results in bowel obstruction, surgical intervention is needed [5, 41].

Abdominal pain post-stenting is typically mild or moderate in severity and can last up to 72 h after stent insertion [5, 56, 57].

Biliary-related complications, although uncommon, have been reported and include biliary obstruction, cholangitis and fistula formation [1, 5, 56]. Patients with tumours in the second segment of the duodenum are particularly vulnerable to biliary obstruction and may require percutaneous transhepatic insertion of metal biliary stents [5, 42]. Management of biliary complications is difficult and varies between individual cases.

### Available Gastroduodenal Stent Designs

Generally, the use of uncovered SEMS is recommended for treatment of gastroduodenal obstructions as they combine low migration rates and avoid covering the ampulla of Vater, thus avoiding biliary obstruction. Uncovered stents are subject to tumour ingrowth. However, as the life expectancy of most patients receiving gastroduodenal stents is short [5, 39, 52], obstruction of uncovered stents is not very common [5, 41, 46]. Stent designs conforming to the duodenal loop are available.

Covered stents resist tumour ingrowth but tend to be more rigid, making them difficult to deploy at distal locations. They have a larger diameter delivery system and are likely to migrate. In addition, they may cause biliary obstruction if they are deployed across the ampulla of Vater [5]. Covered SEMS is indicated for the management of malignant fistulae, perforations or post-surgery leaks (Table 4).

**Table 4:** Complication rates following gastroduodenal stenting

*Major complications of gastroduodenal stenting*	
Procedural mortality	0
Perforation	< 1%
Bleeding	< 1%
*Minor complications*	
Stent obstruction	Up to 17.2%
Stent migration	2.7% uncovered; 10–25% covered
Pain	2.5%
Biliary complications	1.3%

Semi-covered stents are partially covered by a PTFE membrane between two nitinol mesh layers, resulting in lower migration rates, while better resisting tumour ingrowth [1, 5].

## Definitions

- Self-expandable stents
  Tubes that are used to keep a structure (i.e. oesophagus, duodenum, etc.) open to allow the unobstructed passage of food and fluids. These can be either metallic (i.e. stainless steel, nitinol) or plastic and either uncovered, partially covered or fully covered. The latter are coated with polyurethane, polyethylene, polytetrafluoroethylene (PTFE) or silicone according to the manufacturer. Today the majority of self-expandable stents used are metallic (self-expandable metallic stents—SEMS).

- Oesophageal stenting
  The procedure where a self-expanding stent is placed across a narrowed oesophageal segment to allow relief of symptoms, mainly dysphagia.

- Gastroduodenal stenting
  The procedure where a self-expanding metal stent is placed across an intrinsic or extrinsic gastroduodenal obstructing lesion to relieve symptoms.

- Gastric outlet obstruction (GOO)
  Obstructive gastric distension due to impaired emptying of the stomach from an obstructive lesion at the gastric outlet or duodenum (most commonly gastroduodenal cancer).

- Technical success of upper GI stenting
  Defined as successful placement and deployment of the stent across the lesion.

- Clinical success
  Defined as relief of symptoms and/or improvement of oral intake following insertion of SEMS [1, 2, 11].

## Abbreviations

SOP: Standards of practice
SEMS: Self-expanding metal stents
GI: Gastrointestinal
SEPS: Self-expanding plastic stents
CT: Computed tomography
IR: Interventional radiology/radiologist
GA: General anaesthesia
GOJ: Gastro-oesophageal junction
PTFE: Polytetrafluoroethylene
GOO: Gastric outlet obstruction
PLLA: Poly-L-lactic acid
EUS GJ: Endoscopic ultrasound-guided gastrojejunostomy
LAMS: Lumen-apposing metal stents
