# Correction to: CIRSE Standards of Practice on Oesophageal and Gastroduodenal Stenting

**DOI:** 10.1007/s00270-023-03426-w

**Published:** 2023-04-03

**Authors:** Athanasios Diamantopoulos, Shuvro Roy Choudhury, Farah Gillian Irani, Hugo Rio Tinto, Tarun Sabharwal

**Affiliations:** 1 Department of Interventional Radiology, Guy’s and St. Thomas’ NHS Foundation Trust, St Thomas’ Hospital, London, UK; 2 School of Biomedical Engineering & Imaging Sciences, Faculty of Life Sciences & Medicine, Kings College London, London, UK; 3 Radiology, Rabindranath Tagore International Institute of Cardiac Sciences, Kolkata, India; 4 Department of Vascular and Interventional Radiology, Singapore General Hospital, Singapore, Singapore; 5 Radiology Department, Champalimaud Foundation, Lisbon, Portugal

**Correction to: Cardiovasc Intervent Radiol** **https://doi.org/10.1007/s00270-023-03395-0**

This article was originally published under a working title, ‘CIRSE Standards of Practice on Placement of Upper Gastrointestinal Stents’, which was refined during the peer-review process to ‘CIRSE Standards of Practice on Oesophageal and Gastroduodenal Stenting’.

